# Breaking the language barrier: Interpreter service utilisation at Tallaght University Hospital

**DOI:** 10.1007/s11845-026-04324-z

**Published:** 2026-04-21

**Authors:** E. Quinn, A Edwards Murphy, S. A. Talbot, C. Kilkenny, D. Kavanagh, A. Sen Demonte

**Affiliations:** 1 Department of Surgery, Tallaght University Hospital, Tallaght, Dublin 24, Dublin, D24 NR0A Ireland; 2 Department of Surgery, Royal College of Surgeons in Ireland, 123 St. Stephen’s Green, Dublin, D02 YN77 Ireland; 3 Department of Trauma & Orthopaedics, Tallaght University Hospital, Tallaght, Dublin 24, Dublin, D24 NR0A Ireland; 4 Department of Inclusion Health, Tallaght University Hospital, Tallaght, Dublin 24, Dublin, D24 NR0A Ireland

**Keywords:** Health communication, Inclusion health, Interpreter services, Patient safety, Translation services

## Abstract

**Background:**

Effective communication is a cornerstone of equitable healthcare. In multilingual clinical settings, interpreter services play a critical role in ensuring accurate information exchange, patient autonomy, and culturally competent care. Tallaght University Hospital (TUH), is a model 4 hospital serving a diverse urban population, provides a valuable case study on the scale and structure of interpreter utilisation in the Irish healthcare landscape.

**Aims:**

The aim of this study was to describe the utilisation of interpretation services in an Irish model 4 teaching hospital.

**Methods:**

A retrospective analysis was conducted on TUH’s interpreter booking records between January 2024 and June 2025. Data included total bookings, unique patient counts, languages requested, and modality (face-to-face or remote). Figures were stratified by department, with specific attention to the Surgical and Orthopaedic services.

**Results:**

A total of 10,793 interpreter bookings supported 3,397 patients across 64 languages. The majority of bookings were face-to-face interactions (79%) as opposed to remote telephone interpretations (21%). The most requested languages were Ukrainian (3,090 bookings), Russian (1,354 bookings), Polish (1277 bookings), and Romanian (1062) bookings. The Surgical Department accounted for 3,869 bookings (36% of total), serving 1,615 patients with the majority of bookings (3548, 91.7%) occurring in the outpatient setting. The Orthopaedics Department made 579 bookings (5.4% of total), supporting 338 patients, with with the majority of bookings (562, 97.06%) also occurring in the outpatient setting.

**Conclusion:**

Interpreter services are vital for patient safety, informed consent, and professional interactions in a diverse healthcare setting. High utilisation across TUH underscores the need for nationally standardised regulated interpreter services. These findings reinforce the argument for embedding interpretation in national policy and practice within Ireland’s broader inclusion health framework to address disparities experienced by marginalised linguistic communities.

## Introduction

Language is a cornerstone of effective communication and compassionate care in health systems. Recent literature has highlighted how language-discordant care results in compromised trust, incomplete consent, and poorer patient outcomes [1, 2] The 2022 Irish Census reported that over 750,000 people in Ireland speak a language other than English or Irish at home, and total 95,000 residents have limited English proficiency, yet national standards for healthcare interpretation remain lacking [3]. Hurley O’Dwyer et. al appropriately identify that these figures a likely gross underestimate given that over 100,00 Ukrainian citizens have sought temporary protection in Ireland since 2022. This is also alongside an increase individuals seeking international protection applications from other countries, with many of these likely to have limited English proficiency [4].

Given this evolving environment, it is striking that there is little insight into the use of interpretation services in Ireland as no no prior study has quantified interpreter service utilisation in an Irish hospital. It is also worrying in this environment that healthcare professionals are not routinely trained to assess a patient’s linguistic needs or to work effectively with healthcare interpreters. The current system is very dependent on external translation services. It often relies on ad hoc solutions such as untrained family members and friends, or digital tools like Google Translate, which are inappropriate for complex clinical interactions [5]. Compounding this issue, Ireland lacks any regulatory standards for healthcare interpreters—meaning individuals can be employed without formal assessment, accreditation, or training [6]. These low standards not only undermine the work of professionally trained interpreters but also expose patients to significant clinical and ethical risks.

In a multilingual society, the inability to communicate effectively with healthcare professionals can lead to poorer diagnostic outcomes, inadequate treatment adherence, and diminished trust in the healthcare system [7, 8]. This is particularly true for individuals from migrant, asylum-seeking, and minority language backgrounds, including Irish-speaking monolinguals. Ireland’s rapidly evolving demographic profile necessitates a more structured and inclusive approach to healthcare communication.

This study aims to utilise data to assess real-world utilisation trends of interpretation services in an acute model 4 Irish Hospital and to examine its effects as a microcosm of national challenges and opportunities in meeting the needs of an expanding linguistically diverse population. Tallaght University Hospital (TUH) manages a large throughput of patients (e.g. ~ 420,000 attendances annually, 495 adult beds + 67 paediatric beds) and serves a catchment population of ~ 450,000 to 700,000 people. While the socio-demographic area it serves (South & West Dublin) is more ethnically diverse than much of Ireland, it still acts a good setting to explore interpretation services usage in a first analysis of its kind.

## Methods

An initial review of Electronic Medical Records (EMR) was conducted for all General Surgery and Orthopaedics inpatients admitted between 1 st January 2024 and 9th June 2025 to identify non-English-speaking patients and quantify interpreter use. However, due to incomplete or non-standardized documentation of language needs in the EMR, this data was deemed non-representative. As a result, interpreter service booking records from TUH were used as the primary data source for this study, covering the same timeframe**.**

Interpreter booking data from TUH between January 2024 and June 2025 was sourced from the interpretation service and reviewed. This data was anonymized and did not include any individual patient details. All completed bookings were included in the analysis. Data analyzed included total bookings, patient count, face-to-face versus remote interactions, languages booked, and frequency of languages utilised. Bookings were also analyzed by total hospital booking, and across the surgical and orthopedics departments. Unique bookings were classified as patients who had multiple bookings across the timeframe included in the analysis.

## Results

A total of 10,793 interpreter bookings were recorded at TUH between 1 st January 2024 and 9th June 2024 supporting 3,397 unique patients across 64 languages. Of these bookings, 79% (n = 8,514) were delivered face-to-face, while 21% (n = 2,279) were conducted remotely via telephone Table 1. The most frequently requested language during this period was Ukrainian, accounting for 3,090 bookings—representing approximately 28.6% of all interpreter interactions. Russian followed with 1,354 bookings, alongside consistently high demand for Polish (n = 1277), Romanian (n = 1062), and Arabic (n = 778) Table 2.

The Surgical Department generated 36% of total hospital interpreter appointments with 3,869 bookings supporting 1,615 patients. Of these, 3,158 bookings (81.6%) were provided face-to-face, while 390 (12.3%) were conducted remotely. The majority of these carried out in the outpatient setting with 3548 (91.7%) occurring in clinic and just 321 (8.3%) occurring in an inpatient setting. The most frequently requested languages within the surgical department booking were like the total hospital – Ukrainian, Polish, Russian, Romanian, Arabic respectively Table 3.

The Orthopaedics Department accounted for 5.4% of total hospital interpreter appointments with 579 interpreter bookings, supporting 338 patients, with a 128 (22.1%) delivered face-to-face and 434 (74.9%) via remote means. The majority of these carried out in the outpatient setting with 562 (97.06%) occurring in clinic and just 17 (2.94%) occurring in an inpatient setting. The most frequently requested languages within the orthopaedic department booking were similar to the total hospital and surgical department – Ukrainian, Polish, Russian, Romanian, Arabic respectively Table 4.

**Table 1:** Interpreter services booking data – total hospital, surgical department, and orthopaedics department

	**Total Hospital Bookings**	**Surgical Department**	**Orthopaedics Department**
No. of Bookings	10,793	3869	579
Inpatient		321	17
Outpatient		3548	562
No. of patients	3397	1615	338
No. of Face to Face Bookings	8514	3158	128
No. of Remote Bookings	2279	390	434
No. of Language	64	51	30

**Table 2:** Total hospital interpreter service bookings – top 10 languages

Languages	Number of Bookings
Ukrainian	3090
Russian	1354
Polish	1277
Romanian	1062
Arabic	778
Lithuanian	444
Irish Sign Language	274
Chinese (Mandarin)	252
Georgian	201
Portuguese	181

**Table 3:** Surgical department translations services bookings – top 10 languages

Surgical Department Languages	Number of Bookings
Ukrainian	1052
Polish	556
Russian	494
Romanian	414
Arabic	246
Lithuanian	184
Irish Sign Language	89
Chinese-Mandarin/Mandarin Chinese	81
Georgian	78
Portuguese	67

**Table 4:** Orthopaedics department translations services bookings – top 10 languages

Orthopaedics Department Languages	Number of Bookings
Ukrainian	111
Polish	91
Romanian	83
Russian	83
Arabic	40
Lithuanian	38
Irish Sign Language	24
Georgian	20
Somali	17
Portuguese	13

## Discussion

This study provides real-world evidence supporting concerns raised in recent Irish Medical Journal publications about the urgent need for formalised healthcare interpreter services in Ireland [1]. Between January 2024 and June 2025, 10,793 interpreter bookings were made at Tallaght University Hospital (TUH) to support 3,397 patients across 64 languages. Three key findings emerge: (1) a strong preference for face-to-face interpretation, (2) a marked dominance of outpatient utilisation, and (3) substantial linguistic diversity, led by Ukrainian, alongside Polish, Arabic, Romanian, and Russian.

Face-to-face interpretation predominated in high-contact, emotionally sensitive specialties such as Surgery, reflecting the importance of trust, non-verbal communication, and reassurance. Orthopaedics demonstrated greater uptake of remote modalities (approximately 75%), consistent with its typically structured, time-limited outpatient consultations where virtual services may be sufficient. The overwhelming outpatient dominance—91.7% in Surgery and 97.1% in Orthopaedics—indicates that interpreter services are most often used for planned consultations, where communication is central to diagnosis and shared decision-making. However, this pattern may reveal underuse in inpatient care, where language support is equally critical for consent discussions, ward rounds, and multidisciplinary engagement. Barriers such as workflow pressures, booking logistics, and limited staff awareness may contribute to this imbalance, heightening the risk of informal interpretation, compromised confidentiality, and suboptimal care.

This underuse carries significant clinical risk. Inadequate communication during hospital stays can compromise patient understanding, hinder accurate information gathering, reduce engagement with care plans, and affect outcomes—particularly for patients with limited English proficiency. Failure to provide interpreters during inpatient care may result in a reliance on informal supports such as family members or digital translation tools, which are of unvalidated quality, raise ethical concerns and threaten confidentiality [6]. These findings suggest a need for greater integration of interpreter services across the patient journey—not only in structured outpatient settings but also embedded within inpatient workflows. Proactive interpreter involvement, rather than reactive or selective use, is essential to ensure equitable, safe, and person-centred care in a linguistically diverse health system.

The presence of over 60 languages illustrates the depth of linguistic diversity within the hospital’s catchment area which is both sustained and emerging in nature, highlighting the need for scalable, responsive, and professionally supported interpreter services. Demand was highest for Ukrainian with Polish, Arabic, Romanian, and Russian also featured prominently. These demographics reflect both recent migration patterns along with the healthcare needs of long-established minority communities. The demand for translation services will likely increase over the coming years and should challenge healthcare settings to consider in-house interpreters to facilitate timely, accurate, and culturally competent communication across all points of care. Recent data in preprint defining the inclusion health population of TUH has shown that the number of international protection applicants accessing inclusion health services (37.3%) is greater than the displaced Ukrainian population/Beneficiaries of Temporary Protection (13.6%), which indicates that the need for interpreter services spans beyond high-profile migrant groups and must be responsive to the broader, and often more vulnerable, asylum-seeking population with complex healthcare and communication needs [9].

Ireland’s lack of interpreter regulation contrasts with structured systems in comparable jurisdictions. The United Kingdom’s National Health Service maintains accredited interpreter frameworks with standardised training and confidentiality requirements. Canada embeds interpreter provision within its linguistic-rights legislation, supported by provincial accreditation and digital booking platforms. Australia’s National Accreditation Authority for Translators and Interpreters (NAATI) operates a nationally standardised credentialing model integrated into healthcare policy. These systems demonstrate that professional regulation, quality assurance, and clinician education can be effectively institutionalised, improving equity and patient outcomes. Ireland’s current experience—exemplified by TUH—underscores the need for comparable national standards and accountability mechanisms.

These findings align closely with national and international inclusion health frameworks and serve as a microcosm of broader challenges occurring in the healthcare system across Ireland. As highlighted by Hurley O’Dwyer et al., the absence of regulatory standards for healthcare interpreters allows individuals to be employed without formal training, while healthcare professionals often lack guidance on assessing language needs or working effectively with interpreters [1]. This regulatory gap raises significant ethical concerns around confidentiality, accuracy, and informed consent. Breathnach’s study of native Irish speakers reinforces the fundamental principle that patients value receiving care in their preferred language—a need shared by all linguistically minoritised groups [2]. Addressing these gaps requires forward-looking policy action: (1) establish a National Health Interpreter Registry to accredit and regulate practitioners, ensuring consistent standards and accountability; (2) integrate interpreter-use training into healthcare curricula so that clinicians gain competence and confidence in cross-linguistic communication; (3) implement sustainable funding models with ring-fenced interpreter budgets and reimbursement frameworks; and (4) develop a national linguistic-inclusion strategy within healthcare policy, embedding interpreter access across inpatient and outpatient pathways.

This analysis highlights both the strengths and limitations of current interpreter provision in an Irish tertiary hospital. High utilisation, linguistic breadth, and clear preference for face-to-face interpretation demonstrate the value of professional services, yet systemic gaps persist in inpatient use, regulation, and clinician training. Establishing national accreditation, funding, and education frameworks is essential to deliver safe, equitable, and culturally competent care for Ireland’s multilingual population.

## Conclusion

Interpreter services are not auxiliary but central to patient safety, effective communication, and equity in healthcare. Data from Tallaght University Hospital reveal distinct linguistic patterns with direct implications for national policy. Priority actions include the establishment of national accreditation standards for healthcare interpreters, integration of interpreter-use training within medical and nursing curricula, and equitable provision across outpatient and inpatient settings. These steps are critical to embedding language equity and cultural competence within the Irish healthcare system. More broadly, strengthening interpreter provision represents a necessary evolution toward a healthcare model that reflects and serves Ireland’s increasingly multicultural population.
